# A Broad and Potent H1-Specific Human Monoclonal Antibody Produced in Plants Prevents Influenza Virus Infection and Transmission in Guinea Pigs

**DOI:** 10.3390/v12020167

**Published:** 2020-02-02

**Authors:** Jun-Gyu Park, Chengjin Ye, Michael S. Piepenbrink, Aitor Nogales, Haifeng Wang, Michael Shuen, Ashley J. Meyers, Luis Martinez-Sobrido, James J. Kobie

**Affiliations:** 1 Department of Microbiology and Immunology, University of Rochester Medical Center, 601 Elmwood Avenue, Rochester, NY 14642, USA; jungyu_park@urmc.rochester.edu (J.-G.P.); chengjin_ye@urmc.rochester.edu (C.Y.); nogales.aitor@inia.es (A.N.); 2 Department of Medicine, Division of Infectious Diseases, University of Alabama at Birmingham 845 19th Street South, Birmingham, AL 35294, USA; mpiepenbrink@uabmc.edu; 3 Instituto Nacional de Investigación y Tecnología Agraria y Alimentaria, Centro de Investigación en Sanidad Animal (INIA-CISA), 28130 Madrid, Spain; 4 PlantForm Corporation, 1920 Yonge St., Suite 200, Toronto, ON M4S 3E2, Canada; haifeng.wang@plantformcorp.com (H.W.); michael.shuen@plantformcorp.com (M.S.); 5 AntoXa Corporation, 1920 Yonge St., Suite 200, Toronto, ON M4S 3E2, Canada; ashley.meyers@antoxacorp.com

**Keywords:** orthomyxovirus, influenza virus, antivirals, plantibody, prophylactic, therapeutic, monoclonal antibody, antibody treatment, neutralizing antibody, virus infection, virus transmission

## Abstract

Although seasonal influenza vaccines block most predominant influenza types and subtypes, humans still remain vulnerable to waves of seasonal and new potential pandemic influenza viruses for which no immunity may exist because of viral antigenic drift and/or shift. Previously, we described a human monoclonal antibody (hMAb), KPF1, which was produced in human embryonic kidney 293T cells (KPF1-HEK) with broad and potent neutralizing activity against H1N1 influenza A viruses (IAV) in vitro, and prophylactic and therapeutic activities in vivo. In this study, we produced hMAb KPF1 in tobacco plants (KPF1-Antx) and demonstrated how the plant-produced KPF1-Antx hMAb possesses similar biological activity compared with the mammalian-produced KPF1-HEK hMAb. KPF1-Antx hMAb showed broad binding to recombinant HA proteins and H1N1 IAV, including A/California/04/2009 (pH1N1) in vitro, which was comparable to that observed with KPF1-HEK hMAb. Importantly, prophylactic administration of KPF1-Antx hMAb to guinea pigs prevented pH1N1 infection and transmission in both prophylactic and therapeutic experiments, substantiating its clinical potential to prevent and treat H1N1 infections. Collectively, this study demonstrated, for the first time, a plant-produced influenza hMAb with in vitro and in vivo activity against influenza virus. Because of the many advantages of plant-produced hMAbs, such as rapid batch production, low cost, and the absence of mammalian cell products, they represent an alternative strategy for the production of immunotherapeutics for the treatment of influenza viral infections, including emerging seasonal and/or pandemic strains.

## 1. Introduction

Influenza viruses are members of the Orthomyxoviridae family and are responsible for severe respiratory disease in humans [1]. Influenza viruses cause both seasonal epidemics and occasional pandemics of great consequence when novel viruses are introduced into the human population [2]. Although Food and Drug Administration (FDA)-licensed inactivated and live-attenuated influenza vaccines have been used for over seventy years, yearly seasonal influenza viral infections are still responsible for an estimated of 3 to 5 million severe cases of illness and approximately 250,000 to 500,000 annual deaths [1,3,4,5,6]. Influenza virus public health concerns are further aggravated by their ability to efficiently transmit and by the limited available antivirals [7,8,9,10,11]. Influenza viruses are divided into types A, B, C, and D [12,13,14]. Influenza A viruses (IAV) are further classified into different subtypes based on the antigenic surface glycoproteins hemagglutinin (HA; eighteen subtypes) and neuraminidase (NA; eleven subtypes) [1,15,16,17,18]. Among these, HA subtypes are classified into group 1 (H1, H2, H5, H6, H8, H9, H11, H12, H13, and H16–18) and group 2 (H3, H4, H7, H10, H14, and H15) subtypes [15,16,19]. Currently, H1N1 and H3N2 IAV and influenza B viruses (IBV) are circulating in the human population and are responsible for seasonal influenza [1,3]. Among the different influenza viruses, H1N1 IAVs are the most important subtype and have been responsible for causing pandemics with enormous impact on both human health and the economy, as illustrated by the 1918 and 2009 pandemics [1].

Currently, FDA vaccines and antivirals are available for the prevention and treatment, respectively, of influenza viral infections in humans. Influenza vaccines contain viral antigens representing the prevalent H3N2 and H1N1 IAVs as well as one (trivalent) or two (quadrivalent) lineages (Victoria or Yamagata) of IBV currently circulating in humans [20,21,22], and are provided as inactivated or live-attenuated forms. However, although influenza vaccines are able to induce immunity, they have several limitations. These include their lack of effectiveness against drifted seasonal viruses, as recently illustrated by the pandemic H1N1 virus in 2009, the lag time (~2 weeks) to establish an effective immune responses after exposure, and their limited, if any, protection against shifted pandemic viruses. Moreover, yearly formulations are required to protect against constantly changing IAV and/or IBV. In terms of antivirals, three major classes of drugs are FDA-approved for the treatment of influenza infections: NA inhibitors (oseltamivir, zanamivirm and peramivir), matrix protein 2 (M2) inhibitors (amantadine and rimantadine), and polymerase acid (PA) endonuclease inhibitors (Baloxavir, marboxil, or Xofluza) [1,10]. However, influenza antiviral drugs have several limitations, including the lack of antiviral activity of M2 inhibitors against IBV, the emergence of drug resistant variants [23,24,25], and a limited antiviral effect due to rapid metabolism and elimination of the inhibitor [26,27,28,29]. Thus, there is an urgent need to find alternative approaches for the prevention and treatment of influenza infections in humans.

Since 1990, plants have been considered as a potential biofactory for production of biologicals, including monoclonal antibodies (MAbs) [30,31]. MAbs produced in genetically engineered plants (or plantibodies) [32] have been shown to have similar biological activity to mammalian-cell-produced MAbs [33,34]. Because large quantities of MAbs are generally required for passive immunization in vivo, plantibodies represent an excellent option for the production of MAbs for passive immunization, including large-scale manufacturing, low cost, and the absence of animal components [32,34,35,36]. Moreover, plantibodies overcome some of the concerns associated with animal-derived therapeutic MAbs obtained from serum or plasma, including intermediate reactions, pyrogenicity, potential contamination with other zoonotic pathogens and/or toxins, and serum sickness [32,34,35,36,37]. For these reasons, plantibodies have been proposed for the treatment of several bacterial (e.g., Salmonella, Streptococcus, Porphyromonas) and viral (e.g., Ebola virus, EBOV; Hepatitis B virus, HBV; Human immunodeficiency virus, HIV; Porcine epidemic diarrhea virus, PEDV; Rabies virus, RABV; West Nile virus, WNV) infections or toxins (e.g., ricin and shiga toxin) [35,38,39,40,41,42,43,44,45,46]. However, to date, plantibodies have not been used for the treatment of influenza viral infections.

In a recent study, we described that a human monoclonal antibody (hMAb) KPF1 produced in mammalian human embryonic kidney (HEK293T) cells (KPF1-HEK) showed broadly cross-reactive activity against H1N1 IAV in vitro [19]. This is because KPF1-HEK hMAb recognizes a highly conserved and novel epitope in the HA1 globular head region of H1 IAV with high affinity. Importantly, KPF1-HEK hMAb showed broad and potent neutralizing activity in vitro and prophylactic and therapeutic activity in mice against influenza H1N1 IAV [47]. In this study, we demonstrated that a plant-produced KPF1 hMAb (KPF1-Antx) exhibits broad cross-reactivity and potent neutralizing in vitro and in vivo activity against H1N1 IAV. Importantly, KPF1-Antx hMAb has prophylactic and therapeutic activity and could prevent viral transmission in the well-established guinea pig model of influenza virus infection. Altogether, our results demonstrate, for the first time, the feasibility of using plantibodies for the treatment of influenza viral infections in humans.

## 2. Materials and Methods

### 2.1. Cells and Viruses

Madin–Darby Canine Kidney (MDCK; ATCC CCL-34) and human embryonic kidney (HEK293T; ATCC CRL-3216) cells were maintained in Dulbecco’s modified Eagle’s medium (DMEM; Mediatech, Inc.) supplemented with 5% fetal bovine serum (FBS) and 1% PSG (100 unit/mL penicillin, 100 μg/mL streptomycin, and 2 mM L-glutamine) at 37 °C in a 5% CO_2_ atmosphere [19]. Wild-type (WT) influenza viruses A/Brisbane/59/2007 H1N1 (Brisbane/H1N1), A/ California/04/2009 H1N1 (pH1N1), A/New Caledonia/20/1999 H1N1 (NC/H1N1), A/Puerto Rico/8/1934 H1N1 (PR8/H1N1), A/Wisconsin/629-D02473/2009 H1N1 (WI/H1N1), A/Georgia/F32551/2009 H1N1 (GA/H1N1), and A/Brisbane/10/2007 H3N2 (Brisbane/H3N2) were propagated in MDCK cells as previously described [47,48,49].

### 2.2. Production of KPF1 in HEK293T Cells

The human HEK293T cells were grown at 37 °C to approximately 80% confluence in 10 cm tissue culture dishes using DMEM with 5% Hyclone Fetal Clone II (GE Healthcare Lifesciences, Logan, UT, USA) and 1X antibiotic and antimycotic (Gibco, Grand Island, NY, USA). Expression plasmids containing the heavy- and light-chain sequences for KPF1 or the isotype control were transfected into HEK293T cells using jetPRIME transfection reagent (PolyPlus, New York, NY, USA) as previously described [19]. Media was harvested and replenished three times over 8 days. IgG was purified from culture supernatant using Magne Protein A beads (Promega, Madison, WI, USA) and the elution buffer was exchanged with PBS using Amicon Ultra centrifugal filters (Millipore-Sigma, Cork, Ireland).

### 2.3. Production of KPF1 in Plants

Plant expression vectors were assembled using standard recombinant DNA technology, as previously described [50]. The KPF1-Antx and isotype control antibody expression vectors, which included heavy-chain (HC) and light-chain (LC) genes, were co-expressed with an oligosaccharyltransferase from Leishmania major (LmSTT3D) known to enhance N-glycan occupancy of recombinant proteins, using a similar strategy to that previously described [51]. In addition, a third expression vector was utilized to enhance recombinant protein expression by transiently silencing Argonaute1 (AGO1) and Argonaute4 (AGO4) proteins to minimize post-transcriptional gene silencing (PTGS) [Patent WO 2019/023806 A1].

**Figure 1:**
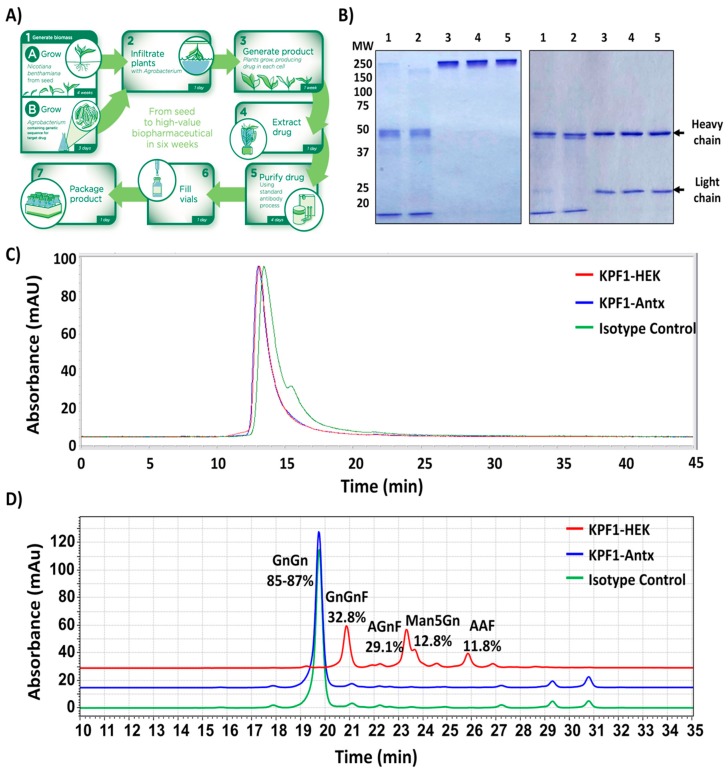
Production and characterization of the KPF1-Antx hMAb. (**A**) Schematic representation of KPF1-Antx hMAb production using the *vivo*XPRESS^®^ platform. Four week old plants were vacuum-infiltrated with a solution containing *Agrobacterium tumefaciens*. The Agrobacteria contained binary vectors for the co-expression of KPF1 or isotype control heavy- and light-chain genes, STT3D, and a system for enhancing protein expression. The infiltrated plants were returned to the greenhouse for 7 days, after which all foliage was harvested and processed to purify KPF1 hMAb using a standard antibody process. (**B**) KPF1-Antx hMAb purification process and analysis: Samples from the KPF1-Antx hMAb purification process: Protein A load (1), protein A flow through (2), protein A elution (3), CaptoQ FT (4), and final purified KPF1-Antx hMAb (5) were analyzed under non-reducing (left) and reducing (right) conditions using standard SDS-PAGE electrophoresis and Coomassie blue staining. Mobility of the molecular marker (MW) in kDa is shown on the left. (**C**) Size-exclusion high performance liquid chromatography: Purified hMAbs (10 µg) were injected into a TSKgel G3000SWXL column equipped with a TSKgel guard column SWXL (Tosoh Biosciences) using an Agilent 1100 Series HPLC. Agilent LC/MSD ChemStation Edition software was used for integration of data. (**D**) N-glycan analysis of KPF1-Antx and KPF1-HEK hMAbs, and plant-produced isotype control: Glycans were prepared using the GlykoPrep^®^ Rapid N-Glycan Preparation kit (PROzyme) and separated by hydrophilic-interaction liquid chromatography (HILIC) using a TSKgel Amide-80 column (Tosoh Bioscience). Glycan species were identified by relative retention time and quantified using auto-integration of each glycan species peak.

KPF1-Antx was produced as outlined in Figure 1A. Briefly, expression vectors were transformed into *Agrobacterium tumefaciens* strain EHA105 and infiltrated at an OD600 of 0.2 into *Nicotiana benthamiana* plant line KDFX, developed by PlantForm (unpublished) for knockdown of the plant-specific β1,2-xylosyltransferase and α1,3-fucosyltransferase [52]. Plant foliage was harvested 7 days post-infiltration and total soluble protein was extracted. Antibodies were purified using MabSelect Protein A followed by Capto Q according to manufacturer protocols (GE Healthcare, Chicago, IL, USA). Purified antibodies were concentrated and formulated to ≥25 mg/mL in PBS.

### 2.4. Coomassie Blue Staining and Western Blot

Total amounts of 2.5, 1.25, 0.625, and 0.313 μg of KPF1-HEK or KPF1-Antx hMAbs were mixed with loading buffer and phosphate-buffered saline (PBS) to a final volume of 20 μL. After being separated by 12% SDS-PAGE, the gel was stained with Coomassie blue staining solution (0.05% Coomassie brilliant blue R-250, 45% methanol, and 7% acetic acid) overnight at room temperature. Subsequently, the gel was transferred to a nitrocellulose membrane. After blocking with 5% bovine serum albumin (BSA) in PBS containing 0.1% Tween 20 (PBST) at room temperature for 1 h, the membrane was incubated with horseradish-peroxidase-conjugated goat anti-human IgG secondary antibody (Jackson ImmunoResearch Laboratories, PA, USA) at room temperature for another 1 h. The blot was developed with ECL detection reagent (Thermo Fisher, California, CA, USA) in the ChemiDoc MP Imaging System (BioRad, Pennsylvania, PA, USA).

### 2.5. Enzyme-Linked Immunosorbent Assay (ELISA)

Binding of KPF1-HEK or KPF1-Antx hMAbs to H1N1 or H3N2 HA proteins was performed using standard ELISA, as previously described [19]. Briefly, ELISA plates (Nunc Maxisorp, Thermo Fisher Scientific, Grand Island, NY, USA) were coated overnight with 1 µg/mL of the indicated recombinant HA proteins obtained from the Biodefense and Emerging Infectious Research Resources Repository (BEI Resources, Manassas, VA) and incubated with 10-fold serially diluted HEK- or Antx-produced KPF1 hMAbs in PBS (starting concentration of 10 µg/mL). Binding was detected with HRP-conjugated anti-human IgG (Jackson ImmunoResearch, Pennsylvania, PA, USA). Plant-produced isotype control (Isotype-Antx) and mammalian HEK293T-produced isotype control (Isotype-HEK) were included as negative controls. In selected ELISAs, increasing concentrations of urea (ranging from 0 to 8 M) were added and the plates were incubated for 15 min at room temperature prior to detection with anti-IgG-HRP to evaluate avidity.

### 2.6. Immunofluorescence Assay (IFA)

MDCK cells (96 well plate format, 5 × 10^4^ cells/well, triplicates) were inoculated with Brisbane/H1N1, pH1N1, NC/H1N1, PR8/H1N1, WI/H1N1, GA/H1N1, and Brisbane/H3N2 at multiplicity of infection (MOI) of 10, or mock infected. At 8 h post-infection (p.i.), cells were fixed with 10% neutral buffered formalin (NBF; Thermo Fisher Scientific, CA, USA) for 1 h and then stained with 1 μg/mL of KPF1-HEK or KPF1-Antx hMAbs, followed by secondary Alexa Fluor 488-conjugated goat anti-human IgG antibody (Life Technologies, Carlsbad, CA, USA). DAPI (4’,6-diamidino-2-phenylindole, Thermo Fisher Scientific, CA, USA) was used to stain cell nuclei. Fluorescent signal images were acquired under an inverted fluorescent microscope (Olympus, Japan) and analyzed by ImageJ software to measure intensity of the staining (NIH, Bethesda, MD, USA) [53].

### 2.7. Microneutralization Assays (MNAs)

Virus microneutralization assays (MNAs) were performed as previously described [19]. Briefly, KPF1-HEK or KPF1-Antx hMAbs, or IgG1 isotype controls, were 2-fold serially diluted in PBS in 96 well plates (starting concentrations of 200 µg/mL). One hundred plaque forming units (PFUs) of each virus (Brisbane/H1N1, pH1N1, NC/H1N1, WI/H1N1, GA/H1N1, and Brisbane/H3N2) were then added to the hMAb dilutions and incubated for 1 h at room temperature. MDCK cells (96 well plate format, 5 × 10^4^ cells/well, quadruplicates) were then infected with the hMAb-virus mixture for 1 h at room temperature. After viral adsorption, cells were maintained in p.i. medium, with 1 µg/mL of N-tosyl-L-phenylalanine chloromethyl ketone (TPCK)-treated trypsin (Sigma-Aldrich, St. Louis, Missouri, MO, USA), and incubated at 37 °C. Virus neutralization was determined by crystal violet staining at 72 h p.i. The neutralization titer 50 (NT_50_) was determined by a sigmoidal dose response curve (GraphPad Prism, v7.0) [19]. Mock-infected cells and viruses in the absence of the KPF1 hMAbs were used as an internal control.

### 2.8. Hemagglutination Inhibition (HAI) Assays

HAI assays were performed to determine the HA-neutralizing capability of KPF1-Antx and KPF1-HEK hMAbs, as previously described [19]. Briefly, KPF1-Antx or KPF1-HEK hMAbs were 2-fold serially diluted (starting concentration of 200 µg/mL) in 96 well V-bottom plates and mixed 1:1 with four hemagglutinating units (HAU) of Brisbane/H1N1, pH1N1, NC/H1N1, PR8/H1N1, WI/H1N1, GA/H1N1, or Brisbane/H3N2 for 60 min at room temperature. HAI titers were determined by adding 0.5% turkey red blood cells (RBCs) to the virus–hMAb mixtures for 30 min on ice. HAI titers were defined as the minimum amount of hMAb that completely inhibited hemagglutination.

### 2.9. In Vivo Experiments

Four week old female Hartley guinea pigs were purchased from Charles River Laboratory and maintained in the animal care facility at the University of Rochester under specific pathogen-free conditions. All animal protocols were approved by the University of Rochester Committee of Animal Resources and complied with the recommendations in the Guide for the Care and Use of Laboratory Animals of the National Research Council [54]. For viral infections, guinea pigs were anesthetized intraperitoneally (i.p.) with ketamine (30 mg/kg) and xylazine (5 mg/kg) and inoculated intranasally (i.n.) with 10^3^ PFU of pH1N1 in 100 μL of PBS. After viral infection, animals were monitored daily for morbidity (body weight loss) and mortality (survival) (data not shown). To determine the prophylactic efficacy of KPF1-Antx hMAb, guinea pigs in two groups (N = 3/group) were weighed and administered (i.p.) 20 mg/kg of KPF1-Antx hMAb or isotype control hMAb (isotype-Antx), and kept in separated cages. After 6 h, guinea pigs were infected (i.n.) with 10^3^ PFU of pH1N1. One day post-infection (d p.i.), sentinel guinea pigs (N = 3/group) were introduced into the cages of infected guinea pigs and monitored for seven days. Viral replication in nasal washes at 2, 4, 6, and 8 d p.i. was determined by immunofocus assay (fluorescent focus-forming units, FFU/mL) using an anti-NP MAb (HB-65) and a FITC-conjugated anti-mouse secondary Ab (Dako). Geometric mean titers and data representation were calculated using GraphPad Prism, v7.0. For therapeutic efficacy, guinea pigs in two groups (N = 3/group) were infected (i.n.) with 10^3^ PFU pH1N1 and kept in separated cages, followed by administration of KPF1-Antx or isotype-Antx hMAbs at 1 d p.i. One day after administration of hMAbs, sentinel guinea pigs (N = 3/group) were introduced into the cages of infected guinea pigs and monitored for 7 days. Viral replication in nasal washes collected at 2, 3, 5, 7, and 9 d p.i. was determined by immunofocus assays as described above. At the completion of the study, guinea pigs were humanely euthanized by administration of a lethal dose of avertin and exsanguination, and lungs were collected for gross observation. Macroscopic pathology scoring was evaluated using ImageJ software to determine the percent of the total surface area of the lung (dorsal and ventral view) affected by consolidation, congestion, and atelectasis, as previously described [53,55,56].

### 2.10. Statistical Analysis

The one-tailed unpaired Student’s *t*-test was used to evaluate significant differences. Data have been expressed as the mean ± standard deviation (SD) using Microsoft Excel software. Values were considered statistically significant when * *p* < 0.05, ** *p* < 0.01, or no significance (n.s.). All data were analyzed using Prism software version 8.00 (GraphPad Software, California, CA, USA).

## 3. Results

### 3.1. Production of the Human Monoclonal Antibody KPF1 in Tobacco Plants

KPF1-Antx was produced in four week old *N. benthamiana* plants as outlined in Figure 1A. Just one week after infiltration with transgene-carrying *Agrobacterium tumefaciens*, IgG was purified from foliage using Protein A followed by Capto Q to remove impurities including endotoxin. KFP1-Antx was expressed at an average of 650 mg/kg of biomass (*N* = 3) and the overall recovery was 68% with an endotoxin level of 0.4 endotoxin units (EU)/mg. Antibody recovery and quality were monitored throughout the purification process using standard SDS-PAGE and Coomassie blue staining (Figure 1B). IgG can be observed in the Protein A load in Figure 1B in addition to host cell proteins such as RuBisCO, which can account for up to 50% of total soluble proteins in leaves [57]. The final KPF1-Antx product was reduced to two independent bands representing the heavy and light chains (50 and 25 kDa, respectively), with no impurities detected. KPF1-Antx and KPF1-HEK were compared using size-exclusion HPLC analysis (Figure 1C). Area under the curve analysis indicated that KPF1-Antx contained 96.3% monomeric IgG and 3.4% low molecular weight (MW) forms, whereas, KPF1-HEK contained 94.5% monomeric IgG, 3.9% low MW forms, and 1.6% high MW forms. These results indicate a greater purity for the plant-derived KPF1-Antx, which included a polishing step (Capto Q). In addition, N-glycosylation profiles were compared using GlykoPrep^®^ Rapid N-Glycan Preparation kit (PROzyme, Hayward, CA) and separation by hydrophilic-interaction liquid chromatography (HILIC) using a TSKgel Amide-80 column (Figure 1D). KPF1-Antx and the isotype control N-glycan profiles were highly similar, with 85%–87% biantennary N-acetylglucosamine (GnGn). Contrary, KPF1-HEK N-glycan profile contained a mixture of N-glycans typically observed on mammalian glycoproteins, including antibodies (32.8% GnGnF, 29.1% AGnF, 12.8% Man5Gn, and 11.8% AAF).

### 3.2. Reactivity of KPF1-Antx and KPF1-HEK hMAbs In Vitro

**Figure 2:**
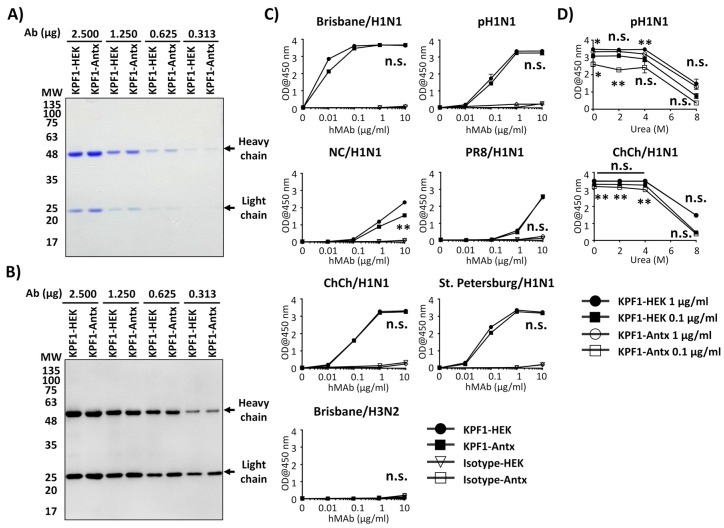
In vitro characterization of KPF1-Antx hMAb. (**A,B**) Purity and mobility of the KPF1-HEK and KPF1-Antx hMAbs: Equal amount of KPF1 hMAbs (2.500, 1.250, 0.625, and 0.313 μg) generated from either HEK293T cells (KPF1-HEK) or tobacco plants (KPF1-Antx) were separated by SDS-PAGE and evaluated by Coomassie blue staining (A) or Western blot using an anti-human IgG HRP-conjugated Ab (B). (**C**) Recombinant HA binding of KPF1-HEK and the KPF1-Antx hMAbs: Binding to 1 μg /mL of indicated HA proteins was determined for serially diluted hMAbs by ELISA. n.s.: no significance. (**D**) Avidity: Binding of hMAbs in the presence of increasing concentrations of urea was determined by ELISA. The statistical analysis between KPF1-HEK and KPF1-Antx (markers above the lines; between 1 μg/mL of KPF1-HEK and KPF1-Antx, marker below the lines: between 0.1 µg/mL of KPF1-HEK, and KPF1-Antx), * *p* < 0.05, ** *p* < 0.01, or no significance (n.s.).

We initially characterized the KPF1 hMAbs generated from either HEK293T cells (KPF1-HEK) or tobacco plants (KPF1-Antx) in vitro (Figure 2). Serially 2-fold diluted (2.5 µg to 0.313 µg) KPF1-HEK and KPF1-Antx hMAbs showed similar characteristics by SDS-PAGE (Figure 2A) and Western blot (Figure 2B), regardless of mammalian or plant production. A comprehensive binding assessment of KPF1-Antx and KPF1-HEK hMAbs to various IAV HA proteins, including Brisbane/H1N1, pH1N1, NC/H1N1, PR8/H1N1, A/Christchurch/16/2011 H1N1 (ChCh/H1N1), A/St. Petersburg27/2011 H1N1 (St. Petersburg/H1N1), and Brisbane/H3N2 was performed (Figure 2C). As expected, KPF1-Antx hMAb bound only H1 Has, including the more recent ChCh/H1N1 and St. Petersburg/H1N1, and binding of KPF1-Antx hMAb to different IAV H1s was similar to that of KPF1-HEK hMAb (Figure 2C). To evaluate the stability of the binding, two different concentrations (0.1 and 1 µg/mL) of KPF1-Antx and KPF1-HEK hMAbs were treated with increasing concentrations of urea (Figure 2D). Both KPF1-Antx and KPF1-HEK hMAbs maintained similar binding affinity in 4 M urea, and substantially diminished in 8 M urea (Figure 2D). These results indicate similar biological properties of the mammalian- and plant-produced KPF1 hMAbs in vitro.

### 3.3. Cross-Reactivity of KPF1-Antx hMAb to H1N1 IAV

**Figure 3:**
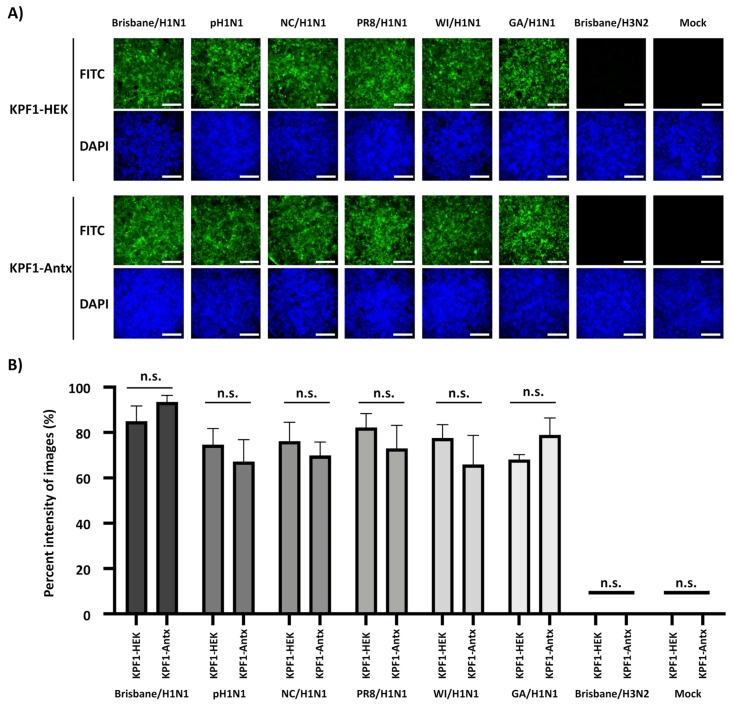
Cross-reactivity of KPF1-HEK and KPF1-Antx hMAbs against IAV H1N1 strains. (**A**) IFA: MDCK cells were infected (MOI 10) with Brisbane/H1N1, pH1N1, NC/H1N1, PR8/H1N1, WI/H1N1, or GA/H1N1; and Brisbane/ H3N2 influenza A virus; or mock infected. At 12 h p.i., cells were fixed and the cross-reactivity of the KPF1-HEK (top) or KPF1-Antx (bottom) hMAbs was evaluated by IFA. DAPI was used for nuclear staining. Scale bar = 100 µm. (**B**) Cross-reactivity intensity: Images in panel A were analyzed for the intensity of fluorescence using ImageJ. n.s.: no significance.

To characterize the ability of the KPF1-HEK and KPF1-Antx hMAbs to recognize native IAV HA proteins, MDCK cells were infected (MOI 10) with Brisbane/H1N1, pH1N1, NC/H1N1, PR8 /H1N1, WI/H1N1, GA/H1N1, or Brisbane/H3N2 and binding was evaluated and quantified by IFA (Figure 3). Both KPF1-Antx and KPF1-HEK hMAbs recognized all H1N1 IAVs, but not Brisbane/H3N2- or mock-infected cells (Figure 3A). Quantifying the KPF1-HEK and KPF1-Antx hMAb binding reactivity to infected MDCK cells revealed that KPF1-Antx hMAb showed similar levels of recognition and binding to H1N1-infected MDCK cells as that observed with KPF1-HEK hMAb (Figure 3B). This result indicated that KPF1-Antx hMAb possesses similar broad cross-reactivity and ability to recognize HA from H1N1 IAV-infected MDCK cells as those observed with the mammalian HEK-produced KPF1 hMAb.

### 3.4. Broad Neutralization and Hemagglutination Inhibition Activity by KPF1-Antx hMAb

We next evaluated the ability of KPF1-Antx hMAb to neutralize a broad range of H1N1 IAV, similarly to the process previously described with KPF1-HEK hMAb [19]. Both KPF1-Antx and KPF1-HEK hMAbs showed similar cross-reactivity, as assessed by IFA, against different H1N1 viruses (Figure 3A). Importantly, quantification of the IFA results indicated that the ability of KPF1-Antx hMAb to recognize the IAV H1 in infected cells was not statistically different than that of KPF1-HEK hMAb (Figure 3B). Likewise, KPF1-Antx hMAb was able to similarly neutralize Brisbane/H1N1, pH1N1, NC/H1N1, WI/H1N1, and GA/H1N1 strains (NT_50_ KPF1-Antx = 0.195 to 0.780 μg/mL; NT_50_ KPF1-HEK = 0.195 to 1.106 μg/mL), while neutralization of PR8/H1N1 was slightly less efficient (NT_50_ KPF1-Antx = 12.5 μg/mL; NT_50_ KPF1-HEK = 25.0 μg/mL) (Table 1). As expected, KPF1 hMAbs were not able to neutralize Brisbane/H3N2 IAV, even at the highest concentration (200 μg/mL). Both KPF1-Antx and KPF1-HEK hMAbs also showed similar HAI activity against H1N1 IAV and were potent for Brisbane/H1N1, pH1N1, WI/H1N1, and GA/H1N1(HAI = 0.271 to 1.106 μg/mL) but were less efficient for NC/H1N1 and PR8/H1N1 (HAI = 8.714 to 17.425 μg/mL) strains (Table 1).

### 3.5. Prophylactic Activity of KPF1-Antx hMAb In Vivo

**Figure 4:**
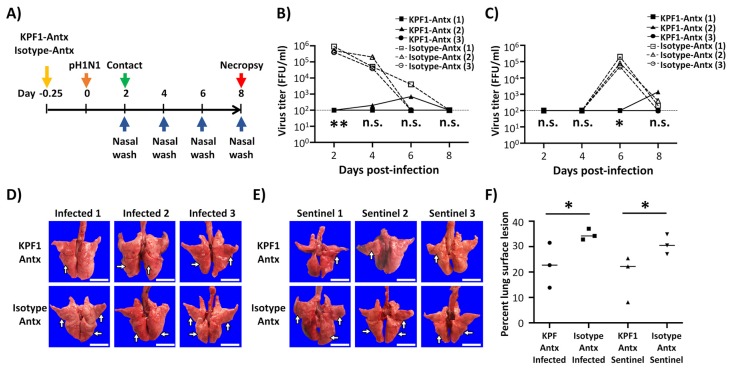
Prophylactic activity of KPF1-Antx hMAb in guinea pigs against pH1N1. (**A**) Schematic representation of the experimental approach: Female Hartley guinea pigs (*n* = 3) were treated (i.p.) with 20 mg/kg of KPF1-Antx hMAb, or with 20 mg/kg of an IgG isotype control (isotype-Antx). Six h post-treatment, guinea pigs were infected (i.n.) with 10^3^ PFU of pH1N1 and monitored daily for 8 days. At 2 d p.i., sentinel guinea pigs were introduced into the same cage of infected guinea pigs, allowing direct contact between the animals. (**B,C**) Viral titers from nasal washes: Viral titers in the nasal washes of infected (B) and sentinel (C) guinea pigs were determined at 2, 4, 6, and 8 d p.i. by IFA (FFU/mL). The dotted line represents the limit of detection of the assay. * *p* < 0.05, ** *p* < 0.01, or no significance (n.s.). (**D**,**E**) Gross observation of lung pathology: All animals were euthanized at 8 d p.i. and lungs were collected from infected (D) or sentinel (E) guinea pigs to observe gross pathological changes such as congestion and atelectasis (arrows). Scale bars = 1 cm. (**F**) Macroscopic pathology scoring: Distributions of pathologic lesion such as consolidation, congestion, and atelectasis were measured using ImageJ and are represented as the percent of the total lung surface area (%). * *p* < 0.05, or no significance (n.s.).

To evaluate the protective efficacy of KPF1-Antx hMAb, Hartley guinea pigs (N = 3/group) received 20 mg/kg of either KPF1-Antx or isotype-Antx control hMAbs 6 h prior to infection with 10^3^ PFU of pH1N1 (Figure 4A). Sentinel guinea pigs (N = 3/group) were put in contact with the infected guinea pigs at 1 d p.i. to assess viral transmission. In the KPF1-Antx hMAb-treated group, only one infected guinea pig and its sentinel guinea pig showed restricted viral infection and shedding, respectively, in the nasal washes (Figure 4B,C). In contrast, all infected guinea pigs treated with the isotype-Antx hMAb control showed higher levels of viral replication (Figure 4B). We also observed viral shedding that resulted in efficient transmission to sentinel guinea pigs in all animals treated with the isotype-Antx hMAb (Figure 4C). Importantly, gross pathology supported the idea that KPF1-Antx hMAb has prophylactic activity (Figure 4D,F). Both infected and sentinel guinea pigs in the KPF1-Antx hMAb treated group showed mild or no multifocal consolidation, congestion, and atelectasis in middle and caudal lobes (Figure 4D,E, respectively), while the isotype-Antx hMAb control-treated group infected (Figure 4D) and sentinel (Figure 4E) guinea pigs showed more severe pathology. Distributions of pathologic lesions on the lung surfaces were measured and compared, and supported the gross observation that the KPF1-Antx hMAb-treated group showed lower lesion values (13.85% to 31.51%) than those of the isotype-Antx control hMAb group (32.81% to 37.05%) (Figure 4F).

### 3.6. Therapeutic Activity of KPF1-Antx hMAb In Vivo

**Figure 5:**
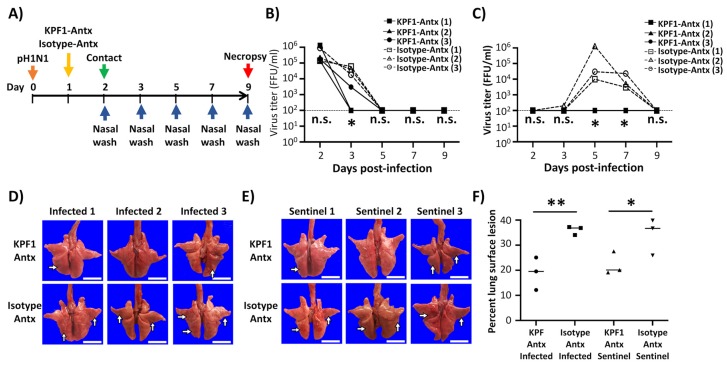
Therapeutic activity of KPF1-Antx hMAb in guinea pigs against pH1N1. (**A**) Schematic representation of the experimental approach: Female Hartley guinea pigs (*n* = 3) were infected (i.n.) with 10^3^ PFU of pH1N1 and treated (i.p.) with 20 mg/kg of KPF1-Antx hMAb, or with 20 mg/kg of an IgG isotype control (Isotype-Antx) 24 h after infection. At 2 d p.i., sentinel guinea pigs (*n* = 3) were introduced into the same cages as infected guinea pigs, allowing direct contact between the animals. (**B**,**C**) Viral titers in nasal washes: To measure viral titers in the upper respiratory tract, nasal washes of infected (B) and sentinel (C) animals were collected on 2, 3, 5, 7, and 9 d p.i. Viral titers were determined by IFA (FFU/mL). * *p* < 0.05, or no significance (n.s.). (**D**,**E**) Gross observations of lung pathology: All animals were euthanized at 8 d p.i. and lungs were collected from infected (D) or sentinel (E) guinea pigs to observe gross pathological changes such as congestion and atelectasis (arrows). Scale bars = 1 cm. (**F**) Macroscopic pathology scoring: Distributions of pathological lesions, including consolidation, congestion, and atelectasis, were measured using ImageJ and have been represented as the percent of the total lung surface area (%).* *p* < 0.05, ** *p* < 0.01, or no significance (n.s.).

To assess the therapeutic activity of the KPF1-Antx hMAb, guinea pigs (N = 3/group) were infected with 10^3^ PFU of pH1N1 and then treated, 24 h p.i., with 20 mg/kg of either KPF1-Antx or isotype-Antx control hMAbs. Sentinel guinea pigs (N = 3/group) were put in contact with the infected guinea pigs at 2 d p.i. (Figure 5A) and evaluated for viral infection and shedding by determining viral titers in the nasal washes (Figure 5B,C, respectively). We observed high viral titers in infected guinea pigs treated with KPF1-Antx and the isotype-Antx control hMAbs at 2 d p.i. However, from 3 d p.i., the KPF1-Antx hMAb-treated group showed lower levels of viral replication in one of the infected guinea pigs and no viral shedding in the two other infected guinea pigs as compared with the isotype-Antx-treated control group (Figure 5B). Importantly, no virus was detected in the sentinel guinea pig group that were put in contact with the infected KPF1-Antx hMAb (Figure 5C). As expected, gross pathological observations of the KPF1-Antx hMAb-treated group showed only mild locally extensive congestion and atelectasis in caudal lobes and surface lesion scores ranging from 12.13% to 25.07% (Figure 5F), while guinea pigs in the isotype-Antx hMAb treated group had more severe and larger distribution of pathological lesions affecting the middle and caudal lobes, and surface lesion scores ranging from 34.03% to 37.20% (Figure 5F). Altogether, these results demonstrate that the plant-produced KPF1-Antx hMAb has both prophylactic and therapeutic activity against pH1N1, including the ability to prevent viral transmission, in guinea pigs.

## 4. Discussion

To date, FDA-licensed vaccines and antivirals are the most effective methods available to prevent and/or control influenza viral infections. However, influenza vaccines require at least 2 weeks after administration to induce protective immune responses against viral infection. In the case of antivirals, their effectiveness is dependent on being administered within 48 h after the appearance of symptoms [58,59]. Importantly, antiviral-resistant strains have been described [1,23,24,25]. Consequently, these prevention and treatment methods still leave substantial public health vulnerabilities.

In a previous study, we described a HEK293T-produced KPF1 hMAb (KPF1-HEK) that showed broad binding and neutralizing activity against H1N1 IAV isolates in vitro and potent prophylactic and therapeutic activities in vivo [19], because of its ability to bind to a conserved residue in the H1 hemagglutinin globular head of IAV [19]. In our current study, we examined the efficacy of a plant-produced KPF1 hMAb (KPF1-Antx) that possessed similar in vitro properties to KPF1-HEK hMAb and prevented influenza infection and transmission in guinea pigs. KPF1-Antx was produced in *N. benthamiana* plants using the vivoXPRESS^®^ platform (Figure 1A), where four week old plants were infiltrated with Agrobacteria containing expression vectors. Purified hMAb was recovered just 7 days after infiltration from the foliage using standard antibody purification techniques. SDS-PAGE (Figure 2A) and SEC-HPLC (Figure 1C) revealed that KPF1-Antx and KPF1-HEK were highly similar. KPF1-Antx was engineered to have predominantly one glycan species—GnGn (Figure 1D). KPF1-Antx hMAb bound various recombinant and native H1 Has, as determined by ELISA and IFA assays (Figure 2 and Figure 3, respectively). Importantly, KPF1-Antx hMAb showed neutralization and HAI activities similar to those of KPF1-HEK hMAb (Table 1), highlighting that KPF1-Antx hMAb possesses and maintains similar avidity and affinity properties as the mammalian-produced KPF1 hMAb. Notably, judging by the potent binding affinity for recent isolates, such as ChCh/H1N1 and St. Petersburg27/H1N1 (Figure 2), KPF1 hMAb covers a substantial antiviral breadth, including recent H1 isolates (Figure 3).

We also examined the activity of KPF1-Antx hMAb to protect (prophylactic) and treat (therapeutic) pH1N1-infected guinea pigs, as well as to prevent direct contact transmission (Figure 4 and Figure 5, respectively). KPF1-Antx hMAb showed potent prophylactic and therapeutic activity against pH1N1 infection in guinea pigs (Figure 4 and Figure 5, respectively), consistent with its in vitro antiviral and HAI properties. Moreover, administration of KPF1-Antx hMAb blocked transmission of pH1N1 infection to sentinel guinea pigs from direct contact with infected animals (Figure 4 and Figure 5, respectively).

Previous studies have described tobacco-derived plantibodies that showed potent therapeutic activity against multiple viruses such as EBOV, HBV, HIV, PEDV, RABV, WNV, and others in small animal models of infection, e.g., mice [39,42,43,44,45,46]. However, to our knowledge, this is the first study demonstrating the ability of plantibodies to protect against influenza viral infections. Concerns exist that plantibodies can be immunogenic and/or allergenic in animals and humans because of a non-mammalian glycosylation pattern [60]. In view of that, a proprietary *N. benthamiana* plant line, KDFX, with knock-down of β1,2-xylosyltransferase and α1,3-fucosyltransfere, was used to reduce the addition of plant-specific glycan species. As a result, no plant-specific sugars were detected (Figure 1D). In the future, the vivoXPRESS^®^ platform could be used to tailor the N-glycan profile (i.e., addition of galactose and/or α1,6-fucose). Follow-up studies to directly and extensively compare mammalian-produced to plant-produced influenza specific antibodies for the impact of potential N-glycan differences on their activity or pharmacodynamics could be done to discern this further. Notably, none of the hMAb KPF1-Antx- or isotype-Antx-treated guinea pigs showed any adverse side effects and/or clinical signs in any of the in vivo experiments at a dose of 20 mg/kg.

Altogether, we substantiated the clinical potential of KPF1 to prevent and treat H1N1 infection in a second animal model, the guinea pig, including its potent ability to prevent transmission, and, for the first time, demonstrated the feasibility of using a plant-produced hMAb for the prophylactic and therapeutic treatment of influenza infections. Plant-produced hMAbs could represent an excellent option for the treatment and control of influenza viruses against which vaccines are not effective (e.g., seasonal) or available (e.g., pandemic), or for which FDA-approved antivirals are ineffective. Moreover, our results also suggest the feasibility of implementing hMAbs produced in tobacco plants for the treatment of other viral infections. However, further investigation will be needed to properly evaluate the safety and efficacy of using such plant-produced hMAbs in humans.

## Figures and Tables

**Table 1:** MNA and HAI assays with KPF1-HEK and KPF1-Antx hMAbs.

Viruses	NT_50_ (µg/mL) MNA		HAI (µg/mL)	
	**KPF1-HEK**	KPF1-Antx	KPF1-HEK	KPF1-Antx
**Brisbane/H1N1**	0.708	0.780	0.552	0.552
**pH1N1**	0.427	0.427	1.106	0.552
**NC/H1N1**	1.106	0.715	8.714	17.425
**PR8/H1N1**	25.0	12.5	17.425	17.425
**WI/H1N1**	0.195	0.195	0.271	0.271
**GA/H1N1**	0.353	0.221	0.271	0.271
**Brisbane/H3N2**	>200	>200	ND	ND
